# Weathering-Resistant Replicas Fabricated by a Three-Dimensional Printing Robotic Platform Induce Shoaling Behavior in Zebrafish

**DOI:** 10.3390/s22093481

**Published:** 2022-05-03

**Authors:** Wei-Lin Wu, Di-Ching Li, Yen-Shuo Chen, Fu-Hsiang Ko

**Affiliations:** Department of Materials Science and Engineering, National Yang Ming Chiao Tung University, 1001 University Road, Hsinchu 30010, Taiwan; lin110489@gmail.com (W.-L.W.); ldc000756@gmail.com (D.-C.L.); rubioibur00@gmail.com (Y.-S.C.)

**Keywords:** *Danio rerio*, shoaling, 3D-printed replica, weathering resistance

## Abstract

In recent decades, zebrafish have become an increasingly popular laboratory organism in several fields of research due to their ease of reproduction and rapid maturation. In particular, shoaling behavior has attracted the attention of many researchers. This article presents a fully printed robotic model used to sense and stimulate shoaling behavior in zebrafish (*Danio rerio*). Specifically, we exposed laboratory-fabricated replicated materials to critical acid/base/salt environments and evaluated the mechanical, optical, and surface properties after a three-month immersion period. Focusing on weatherability, these test samples maintained high tensile strength (~45 MPa) and relatively similar transmission (>85%T in the visible region), as determined by UV–vis/FTIR spectroscopy. Three-dimensional (3D) printing technology allowed printing of models with different sizes and appearances. We describe the sense of zebrafish responses to replicas of different sizes and reveal that replicas approximating the true zebrafish size (3 cm) are more attractive than larger replicas (5 cm). This observation suggests that larger replicas appear as predators to the zebrafish and cause fleeing behavior. In this study, we determined the weatherability of a high-transparency resin and used it to fabricate a fully printed driving device to induce shoaling by zebrafish. Finally, we demonstrate a weathering-resistant (for three months) 3D-printed decoy model with potential utility for future studies of outdoor shoaling behavior, and the result has the potential to replace the traditional metal frame devices used in outdoor experiments.

## 1. Introduction

In recent decades, zebrafish (*Danio rerio*) has become an increasingly popular laboratory model organism, not only in the field of genetics [1,2], but also in behavioral neuroscience [3]. The increasing use of zebrafish in animal experiments is due to several factors, including not only ease of breeding but also rapid development from the larval to adult stage [4,5]. Moreover, zebrafish possess some human-like properties such as gene sequence and social behavior. Although zebrafish have become common vertebrata in the laboratory, studies on zebrafish behavior are far fewer than genetic and pharmacological investigations [6]. This situation has changed in recent years. Attention has been given to the learning capabilities of adult fish [7], larval zebrafish behavioral responses [8], and behavior-related disease models. However, studies on shoaling remain rare. The most well-known social behaviors of zebrafish, shoaling and schooling, were defined in 1986 [9]. Both behaviors help fish increase their survival/reproduction rates. Furthermore, approximately half of all fish species shoal in their lives. Scientists attribute fish shoaling behavior to reasons including antipredatory effects and foraging promotion. Regarding antipredatory effects, a large shoal can detect predators sooner than a single fish. Moreover, when living in a large shoal, an individual fish would be less likely to be caught since the predator may be confused, and in a number of shoals (e.g., three), one would not be targeted easily. For the purpose of foraging, it is easier for a single fish to find food when in a group of fish. When one fish detects food, it attracts other members to this food.

Various biologically inspired robotic replicas have been widely used in behavioral research; this technique has been demonstrated to be an effective and reproducible tool to study the group behavior of various organisms, including insects [10] and reptiles [11]. In early laboratory experiments, scientists put more effort into rats and mice [12]. After the genome of zebrafish was fully sequenced [13], researchers found that this organism has several characteristics similar to those of humans [14]. Among the studies of zebrafish behavior, some researchers have tried to replace live fish with replicas. In contrast with classical protocols based on live fish stimulation, the robotic replicas showed stable and repeatable motion trajectories, which is beneficial for systematizing the behavior of focal individuals [15]. In previous work [16,17], several research teams reported the combination of robotic replicas and three-dimensional (3D) printing. In these studies, it was proposed that the three axes of motion could be controlled by three independent motor control platforms, allowing for more precise control. We used 3D printing to prepare our system for facile part size adjustment and assembly testing. Three-dimensional printing technology has many advantages for customization and laboratory fabrication. In the past, many studies were devoted to improving the performance of printing resins, whether from microstructure [18] or material doping [19,20], and this technology has advanced greatly. The various methods of 3D printing include fused deposition modeling (FDM), digital light processing (DLP), and stereolithography. To obtain relatively excellent mechanical strength [21], we used a commercial stereolithography appearance (SLA) 3D printer to fabricate our samples. Finally, we manufactured a fully printed tracing system and different appearance replicas for behavioral experiments. Previous work has shown that zebrafish are more attracted to live fish than replicas [22]. Moreover, one paper used a transparent board to separate live stimuli from the focal fish [23]. Such isolation enables a two-way interaction between the focal fish and the stimulus during the experiment. However, in this study, we separated live stimuli and focal fish with unidirectional glass to reduce the visual interaction of live stimuli with focal fish. In prior methods [24], 3D printing was typically used only for replica printing, while the supporting frame was mostly made of metal. However, special treatments are required in corrosive or humid environments, otherwise, the system is prone to rusting and damage. As there are plans to conduct outdoor shoaling experiments in the future, 3D printing is better than metal for customization, and the metal can be partially replaced by printing parts at low cost. To prove the sturdiness of the materials, we tested the mechanical strengths and actually printed the parts and assembled the finished product to be used for experiments. Finally, the weather resistance experiment conducted in this research proved that if our material is in the sea or in some acidic or alkaline surroundings, e.g., acid rain, it can still be used for a long time. Furthermore, we demonstrated the interaction between different outlook replicas and focal fish behavior.

## 2. Experimental Section

### 2.1. Subjects

Zebrafish (adults, 6–7 months old) were purchased at a local hypermarket (Yuzhongyu aquarium hypermarket, Hsinchu, Taiwan). Twelve zebrafish were placed in a 20 L water tank for 28 days before the experiment. In the zebrafish shoaling assay, there are a number of feeding conditions that affect physiological state and shoaling behavior, such as temperature, circadian rhythm [25], and feeding schedule [26]. In our work, the temperature of the habitat water was maintained at approximately 26 °C, and the fluorescent lights were set on a regular circadian rhythm (light on at 9:00, light off at 20:00). Additionally, zebrafish were fed between 14:00 and 15:00.

### 2.2. Robotic Platform

**Figure 1:**
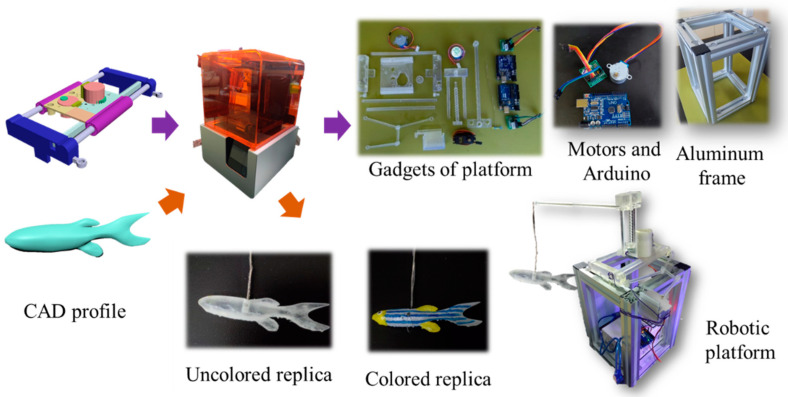
Manufacturing flow for the robotic and sensing platform.

**Figure 2:**
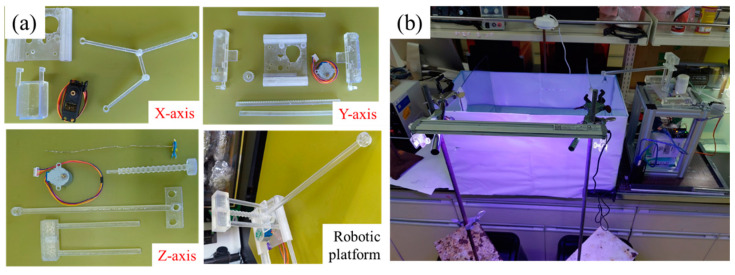
(**a**) Homemade robotic components for different axes. (**b**) The homemade system for evaluating shoaling behavior of zebrafish.

Figure 1 shows the manufacturing flow for the robotic platform. The 3D printing platform components and robotic replicas were designed in the computer-aided design (CAD) software AutoCAD and printed in clear resin (poly (methyl methacrylate), PMMA) using a stereolithography appearance (SLA) 3D printer Form 3 (FormLabs Co., Somerville, MA, USA). Clear resin with high transmission in the visible region was provided by Formlabs Co. The main components of the resin are urethane methacrylate (55~75%) and methyl methacrylate monomer (15~25%). After postcuring with a 405 nm LED light with a power of 1.25 mW/cm^2^ for 60 min at 60 °C, the printing resin had a mechanical strength comparable to 45 MPa. To create customized and versatile replicas, 3D printing has been a promising technique in past research [17]. Accordingly, we used 3D printing to help us fabricate the platform and gadgets. Earlier studies used robots that followed two-dimensional trajectories that either were impractical or had limited mobility in response to stimuli [16,27]. To improve on the imitation lacking in previous studies, one team experimented with a three-degree-of-freedom platform [17]. This design enabled 3D-printed replicas to achieve more realistic zebrafish movements. In the present study, we wanted our replicas to move like a live individual, and the moving part of this work was divided into three parts: X, Y, and Z. Each component (shown in Figure 2a) controls the movement of the replica and the entire platform model in one direction.

Aiming for high transparency (~85%) in the visible light region, we chose clear resin to print robotic platform components to ensure that our platform did not affect the behavior of the focal fish. We also compared two different replicas, transparent and colored. In the results, we reveal the attractiveness of the transparent and colored replicas.

In contrast to previous studies, we employed an all-printing approach to prepare our mechanical platform. This method has the advantages of customization, transparency, and 3D freedom. We compare the material usage of the mechanical platform and the freedom of movement of the replica in Table 1.

### 2.3. Weathering Test

First, we printed two different standard samples for the tensile test and other measurements: one was a dog bone shape with a cross-sectional size of 1 mm × 1 mm, and the other was a thin film with a size of 1 cm × 1 cm × 1 mm. Then, we placed them in three different solutions to simulate extreme environments: 1 M nitric acid aqueous solution, 1 M sodium hydroxide aqueous solution, and sea water (3.5% salt solution). After three months of immersion, we removed the experimental samples and performed tensile tests, contact angle measurements, and ultraviolet (UV)–visible and Fourier transform infrared (FTIR) spectroscopic analyses. Furthermore, we verified the weathering stability of our printed products with several physical–mechanical analyses.

In tensile tests, we stretched the dog bone sample with a force gauge (FG-6020SD, Lutron, Taipei, Taiwan) and recorded the maximum tensile strength of the sample at the break point. In addition, we use two solvents with different polarities, DI water and diiodomethane, to calculate surface tension with a contact angle meter (SURFTENS, OEG, Hessisch Oldendorf, Germany). Then, we measured the UV–visible–NIR spectrum with an ultraviolet-visible spectrophotometer (UV-2600i, Shimadzu, Kyoto, Japan) with an integrating sphere. Last, we use an FTIR spectrometer (Vertex 70, Bruker, Billerica, MA, USA) to analyze the optical densities at wavelengths between 2.5 μm and 25 μm.

### 2.4. Shoaling Test

**Figure 3:**
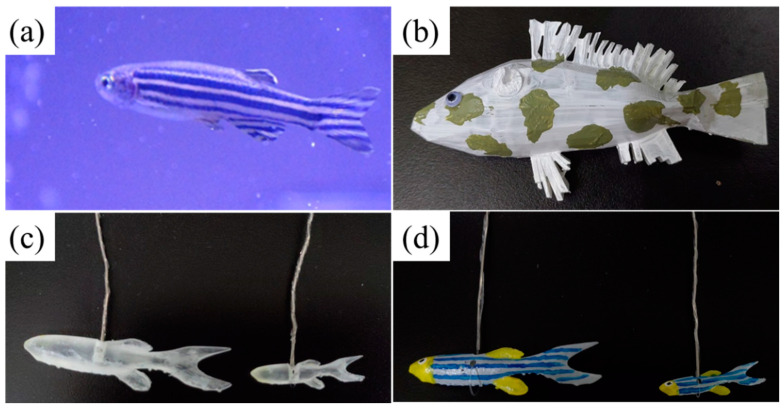
The appearances of (**a**) living zebrafish, (**b**) an Indian leaf fish replica, (**c**) transparent zebrafish replicas of different sizes, and (**d**) colored zebrafish replicas of different sizes.

One tank (60 cm × 30 cm × 36 cm) was used for the experiment. As shown in Figure 2b, we divided the tank into three compartments with two transparent acrylic boards. The lengths of the three compartments were 10 cm, 40 cm, and 10 cm from left to right. The camera was 60 cm above the table surface. Then, we placed the first zebrafish in the central compartment as ‘c’ and another zebrafish in the left compartment as ‘live stimulus’. After 15 min of observation of the focal fish, we removed the live stimulus and placed the 3D-printed zebrafish into the right compartment as a ‘replica’. The appearances of living fish and all replicas used in this experiment are shown in Figure 3. After the start of the motors, we recorded the behavior of the focal fish for 15 min. In the final part, we used the Tracker software to analyze our experimental video.

## 3. Results and Discussion

### 3.1. Weathering Resistance Experiment

In many reports, the degradation of polymers occurs in the absence of oxygen and sunlight, which is called photooxidative degradation [28]. Fundamentally, these reactions occur through a free radical mechanism, similar in some ways to thermal oxidation or chemical reactions. Accordingly, test samples were immersed in a variety of corrosive environments, which resulted in loss of properties (e.g., tensile strength, surface tension, light transmittance)**.** Specifically, the tensile strength was reduced by half [29], which is fatal to our driving skeleton, so a series of tests were carried out to ensure the stability of the material during long-term use. First, we simply exposed our product to outdoor sunshine. In addition, the potential for catastrophic failure that a fully printing system would experience when immersed in acid/alkaline/sea water has become an obstacle for outdoor applications. Furthermore, the degradation process of printing resin is a fundamental but important topic in practice.

The weathering-resistant characteristics of fracture strength, surface tension, and optical properties ensure that our platform and replicas exhibit potential in long-term, outdoor applications. In the first part, we performed a tensile test to characterize the mechanical strength of our printed resin. Second, we performed contact angle measurements and analyzed the surface tension differences among soaking solutions such as acid, alkali, and sea water. Finally, we performed optical spectroscopic analysis, including UV–visible and FTIR measurements, to test the high transparency in the visible region and study the etching mechanism of the sample.

#### 3.1.1. Effect of Sunshine Exposure on the Surface Tension of 3D-Printed Materials

**Figure 4:**
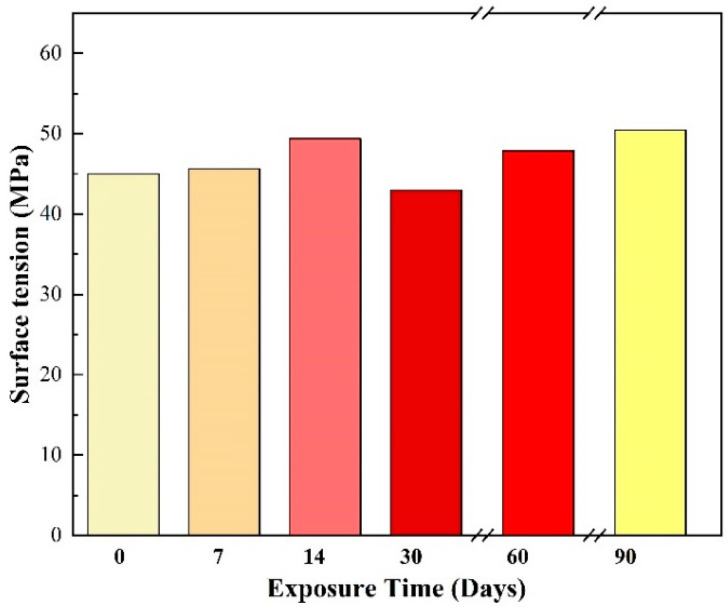
Surface tension of printed materials exposed to natural sunlight for three months.

First, we put our test sample into 3.5% salt water to simulate a sea environment and compared the results after exposure to natural sunlight for different amounts of time, which can help us to understand the degradation stage and material loss of our sample. It is surprising that the test sample did not present an obvious decay (<10%) in surface properties, as shown in Figure 4. This result supports the application potential of our fully printed system in many outdoor scenarios.

#### 3.1.2. Effect of the Soaking Environment on the Surface Tension of 3D-Printed Materials

**Figure 5:**
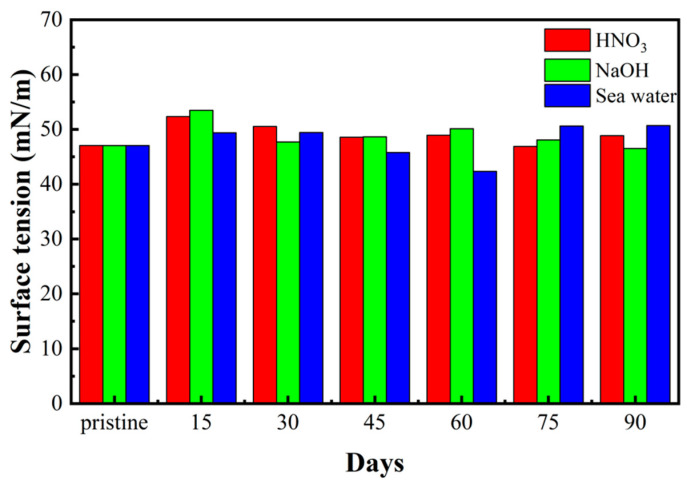
Surface tension of printed materials in different extreme environments after three months.

When a sample reacts with a specific chemical compound, the surface characteristics will change accordingly. Therefore, we performed contact angle measurements and surface tension analyses to determine the surface conditions after three months of immersion. Due to the inertness of strong acid and alkali solutions, similar results were observed in the surface tension with soaking time under extreme conditions. As shown in Figure 5, the surface tension also oscillates in a related small region. The surface tension was found to always be determined by the surface properties, corroborating that the surface of the samples did not react with acid, alkali, or salt, while the results for both surface tension and fracture strength indicated a very slight influence due to sample deterioration.

#### 3.1.3. Effect of Soaking Environment on the Fracture Strength of 3D-Printed Materials

From the results described above, we inferred that the stereolithography resin that we used can reduce photodegradation and maintain good stability under outdoor sunlight exposure. Then, we performed simulations under more extreme conditions to try to find the weather resistance limit of our sample.

**Figure 6:**
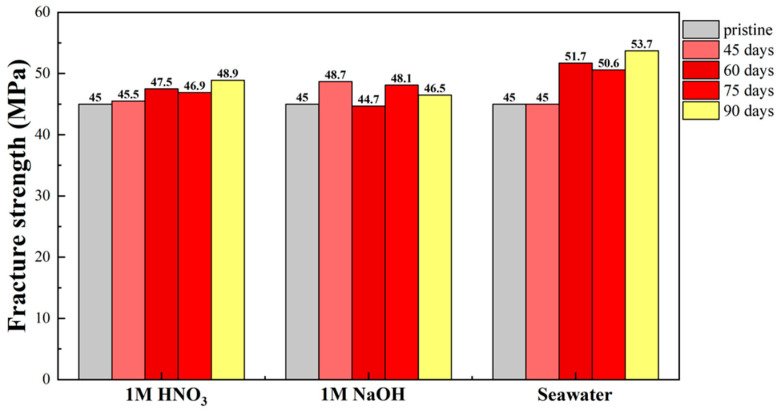
Ultimate tensile strengths of printed materials immersed in different extreme environments for three months.

Since the motion system is supposed to bear the weight of the motor and replica, mechanical tests, especially tensile strength tests, are attractive to us. In a previous report, polypropylene and poly(vinyl chloride) [30] were affected by UV/natural sunlight and presented degraded tensile properties. Therefore, the fact that our test sample maintains its tensile strength when immersed in extreme surroundings is important and of interest to us. Figure 6 shows plots of the fracture strength of the clear resin samples from different soaking environments (acid/alkali/sea water) against soaking time.

From this result, it is seen that the tensile strength of the clear resin does not decrease significantly; indeed, the resin retains its original mechanical properties under three different extreme conditions. Its performance exhibits high stability in several reactive solutions. In other words, the printed products can be used in outdoor experiments over a long period of time because of their excellent mechanical stability in normal territorial waters, even when used in polluted water over the long term.

#### 3.1.4. Effect of Soaking Environment on the Optical Properties of 3D-Printed Materials

**Figure 7:**
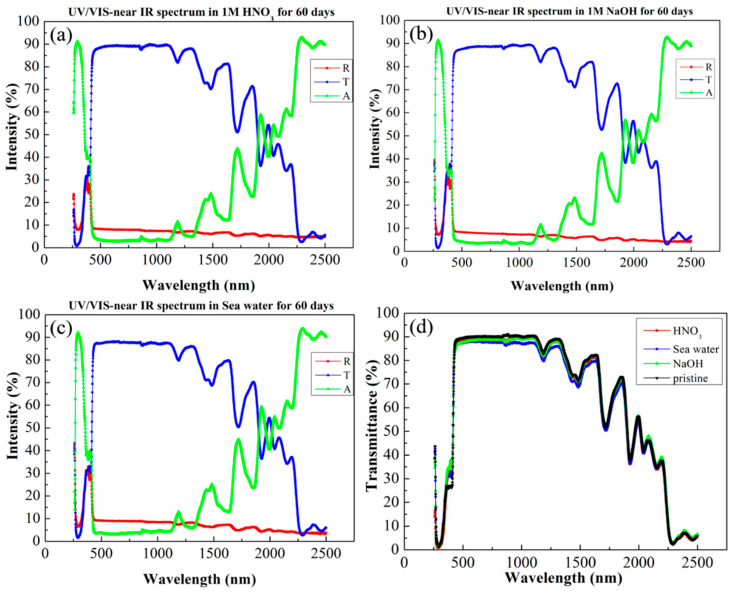
UV–vis–near-IR spectra of printed materials in (**a**) 1 M nitric acid aqueous solution, (**b**) 1 M sodium hydroxide aqueous solution, and (**c**) sea water (3.5% salt solution). (**d**) Transmittance spectra obtained after exposure to different extreme environments for three months.

**Figure 8:**
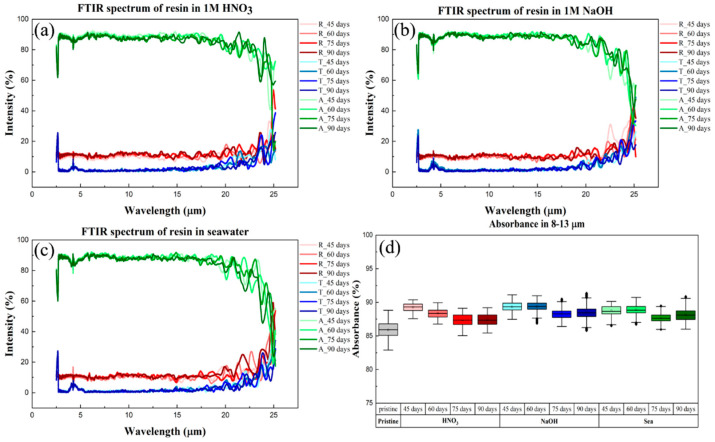
FTIR spectra of printed materials in (**a**) 1 M nitric acid aqueous solution, (**b**) 1 M sodium hydroxide aqueous solution, and (**c**) sea water (3.5% salt solution). (**d**) Absorbance data determined after exposure to different extreme environments for three months.

The effect of different impregnation solutions on our printing resins has already been mentioned in the test results above. While the tensile strength and surface tension can indicate the weatherability of the print, the optical properties need to be evaluated by UV–vis and FTIR spectroscopy. These two techniques are complementary in detecting transmittance in the visible region and the formation of characteristic peaks for carbonyl products. In this case, the UV–vis and FTIR spectra are shown in Figure 7 and Figure 8, respectively.

In Figure 7, we present the UV–visible–NIR spectrum, which shows the reflectance, transmittance, and absorbance of the material at wavelengths between 300 nm and 2.5 μm; these parameters are represented by R, T, and A in the caption. In other studies, photodegradation caused drastic changes in UV absorbance, but this was not in the case for our data, which confirmed that our material did not age and degrade over prolonged use. Next, we wanted to determine the resistance of the material surface to corrosive solutions. Figure 7 shows that the original has high transparency (>85%) in the visible region, and the experimental samples still show the same high transparency as the original after being immersed in three different extreme environments. Fresnel equations indicate that surface erosion will greatly reduce visible light transmittance, and our results verified that there was no loss of transmittance due to surface erosion after immersion in extreme environments. This result can be used as indirect evidence for the integrity of the surface topography. On the other hand, in past reports, it has been mentioned that in the atmospheric window, absorption of light within the 8–13 micron range can lead to passive heat dissipation [31]. Furthermore, we can see from Figure 8 that the absorption properties of the resin samples did not change after three months of immersion. We speculate that this passive cooling mechanism gives our printing resins great potential for outdoor experiments. We supposed that changes in surface topography would affect the light transmittance and absorbance of the specimen, and the uniformity of our sample indicated that the surface topography was not changed by erosion.

### 3.2. Shoaling Experiment with a Robotic Replica Obtained from 3D-Printed Materials

In recent years, the concept of biomimetic replicas has been developed [32] to circumvent the limitations of traditional approaches to induce the social behavior of vertebrates by using repeatable and quantifiable motion controllers. For example, 3D-printed replicas of zebrafish can be used to study the shoaling behavior of individuals. In the experimental part, we placed a focal fish in the middle of the live tank and the robotic replica to the right. We then recorded the movements and converted the video into a preference index (PI). In this section, we introduce an index (PI) to quantify how a visual stimulus attracts or scares focus fish. We divided the central compartment into three equal parts; the length of each section was 16 cm. The PI was defined as TN/(TN+TF), where TN was the time that the focal fish spent in the area close to the stimulus and TF was the time spent in the opposite area. Then, we compared the colored and uncolored replicas.

#### 3.2.1. Transparent Replica Behavior

**Figure 9:**
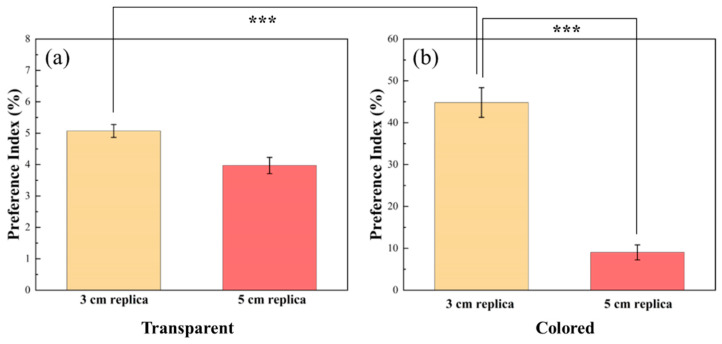
Preference index for (**a**) transparent and (**b**) colored replicas of different sizes (*** indicates *p* < 0.001).

As shown in Figure 9a, the focal fish did not follow the transparent replicas. This situation has been discussed in previous studies because social behavior between two zebrafish relies on their visual systems. Since the zebrafish could not successfully identify transparent replicas, focal fish were not attracted. As a result, the focal fish did not treat the replicas the same as live fish, and shoaling behavior was rare when we looked at the motion trajectories. When we analyzed replicas of different body sizes, there were subtle differences between one size larger and one smaller. From these results, we clearly observed the effects of visual stimuli and zebrafish shoaling behavior.

#### 3.2.2. Colored Replica Characterization

From the above discussion, we propose that transparent zebrafish possess no attractiveness to living zebrafish. Furthermore, we wanted to study the relationship between visual stimuli and shoaling behavior, so we used colored replicas instead and ran the experiment again; see Figure 9b. The PI comparisons demonstrate significant differences between experimental conditions relative to the control group. 

The colored replica shows a PI of approximately 10% compared to 4% with the colorless replica. In the 3 cm replica group, this difference increases. As shown in Figure 9b, the PI of the 3 cm replica with color is more than 40%. On the other hand, the PI is only approximately 5% in the 3 cm replica without color. Through the two figures, we can say that the presence or absence of color substantially affects zebrafish. In addition, 3 cm replicas were more attractive to zebrafish than 5 cm replicas. The PI was much higher in the 3 cm replica with color. As shown in Figure 9a,b, we used the statistical tool *p* value to determine the difference among different appearances. If *p* < 0.05, we may conclude that these data show significant differences. In the chromatic versus achromatic results, the *p* values were less than 0.001, supporting our conclusion about similar sizes and indicating that the colored replicas were more attractive to the zebrafish. Next, we discuss all motion parameters, including average velocity and acceleration.

**Figure 10:**
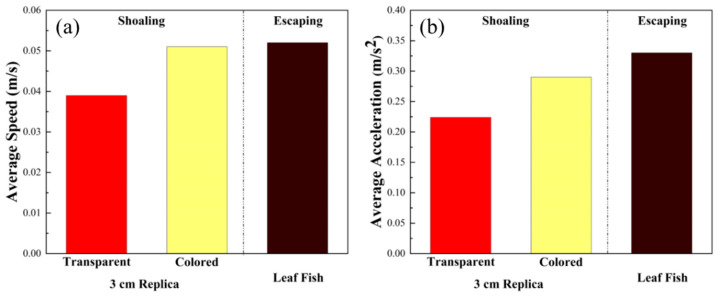
Comparison of (**a**) average speed and (**b**) average acceleration of real fish with transparent and colored replicas sized 3 cm and leaf fish replica (predator).

Figure 10a,b shows that both the average speed and acceleration of the 3 cm replica with color are slightly higher than those of the colorless replica. This result may indicate that although the colored replica is more attractive for zebrafish, they escape with faster speed when they find that it is not a true fish. On the other hand, replicas with no color cannot be identified as similar in the focal fish’s eye. Zebrafish may not perceive it as a companion or predator, thus maintaining a relatively steady speed and acceleration. In Figure 10, we can tell that zebrafish escape the leaf fish replica with faster speed and more acceleration.

Finally, to determine why 3 cm replicas were more attractive than 5 cm replicas, our team supposed that zebrafish would participate in shoaling with fish of sizes equal to or smaller than themselves. Too large a size could make the focal fish try to escape the robotic replica, since large novel objects often mean predators of marine life. These results are similar to those of Bartolini’s team [33], and the difference in average speeds seen in Figure 10a provides indirect evidence for the predator hypothesis. Body size and color have been found to be determinants for attraction of zebrafish. These findings are similar to the results of a previous article [34] indicating that visual stimuli are likely the key points leading to shoaling behavior in zebrafish.

## 4. Conclusions

In this work, we successfully validated the weatherability of our printed samples and proposed an application with which to sense shoaling behaviors in zebrafish. After three months of harsh environmental simulation, the tensile strength of the resin in our sensing samples decreased by less than 10%, the surface energy remained the same as that of the original, and the transmittance of 85% in the visible light region was maintained. Based on the above data, we conclude that our printed material has complete stability in corrosive solutions for three months. Additionally, replicas of the control motion system and robotic platform were all printed by a 3D printer, which was not available in previous studies. We also show that the colored replicas are more attractive than transparent replicas because the former are more visually similar to living stimuli from the fish perspective. Second, when the body length of the replica was the same (3 cm) as that of real zebrafish, the replicas were more attractive. In contrast, most focal fish were not attracted by larger replicas that, in their eyes, were as large as a predator, necessitating escape. Finally, from the average velocity and acceleration results, it can be seen that the zebrafish kept their distance and escaped faster when facing the leaf fish replica. All kits in our sensing system are 3D printed and have the potential for outdoor experiments with similar results through multiple analyses after accelerated burn-in testing.

## Figures and Tables

**Table 1:** Comparison of our replica materials with those from literature reports.

Replica Material	Motion Dimension	Platform Material	Homemade	Reference
PLA	2D	Aluminum	Hard	[16]
PMMA	3D	Aluminum	Hard	[17]
PMMA	2D	Plexiglas rod	Hard	[27]
Stereolithography resin (PMMA)	3D	Stereolithography resin (PMMA)	Easy	This work
